# Improvement for most, but not all: changes in newspaper coverage of mental illness from 2008 to 2019 in England

**DOI:** 10.1017/S204579602000089X

**Published:** 2020-11-05

**Authors:** R. Hildersley, L. Potts, C. Anderson, C. Henderson

**Affiliations:** Institute of Psychiatry, Psychology and Neuroscience, King's College London, London, England

**Keywords:** Epidemiology, mental health, mental illness stigma, schizophrenia

## Abstract

**Aims:**

Time to Change, an anti-stigma programme in England, has worked to reduce stigma relating to mental illness in many facets of life. Newspaper reports are an important factor in shaping public attitudes towards mental illnesses, as well as working as a barometer reflecting public opinion. This study aims to assess the way that coverage of mental health topics and different mental illnesses has changed since 2008.

**Method:**

Articles covering mental health in 18 different newspapers were retrieved using keyword searches on two randomly chosen days of each month in 2008, 2009, 2010, 2011, 2013, 2014, 2016 and 2019. A content analysis approach using a structured coding framework was used to extract information from the articles. Logistic regression models were used to estimate the change in odds of each hypothesised stigmatising or anti-stigmatising element occurring in 2019 compared to 2008 and 2016 with a Wald test to assess the overall significance of year as a predictor in the model. Further logistic regression models were used to assess the association between the diagnosis that an article was about and the odds that it was stigmatising, and whether this relationship is moderated by year of publication.

**Results:**

A total of 6731 articles were analysed, and there was a significant increase in anti-stigmatising articles in 2019 compared to 2008 (OR 3.16 (2.60–3.84), *p* < 0.001) and 2016 (OR 1.40 (1.16–1.69), *p* < 0.001). Of the 5142 articles that specified a diagnosis, articles about schizophrenia were 6.37 times more likely to be stigmatising than articles about other diagnoses (OR 6.37 (3.05–13.29) *p* < 0.001), and there was evidence that the strength of this relationship significantly interacted with the year an article was published (*p* = 0.010). Articles about depression were significantly less likely to be stigmatising (OR 0.59 (0.69–0.85) *p* = 0.018) than those about other diagnoses, while there was no difference in coverage of eating disorders *v.* other diagnoses (OR 1.37 (0.67–2.80) *p* = 0.386); neither of these relationships showed an interaction with the year of publication.

**Conclusion:**

Anti-stigma programmes should continue to work with newspapers to improve coverage of mental illness. However, interventions should consider providing specific guidance and promote awareness of rarer mental illnesses, such as schizophrenia, and evaluation should examine whether reductions in stigma extend to people with all mental illness diagnoses.

## Introduction

Stigma, described as ignorance, prejudice and discrimination (Thornicroft *et al*., 2007), towards people with mental illness contributes to inequality (Phelan *et al*., 2014), excess mortality (Laursen *et al*., 2007; Gissler *et al*., 2013; Starace *et al*., 2018) and affects help-seeking behaviour (Thornicroft, 2008; Henderson *et al*., 2013; Schnyder *et al*., 2017). While there is evidence that mental health stigma in England has lessened since 2008, changes are still needed (Evans-Lacko *et al*., 2014; Henderson *et al*., 2016a, 2020; Robinson and Henderson, 2019). Newspaper coverage is one influence on public perceptions of mental illness: people exposed to positive stories about mental illness online and in print media are more likely to report less stigma (Thornton and Wahl, 1996; Corrigan *et al*., 2005, 2013; Klin and Lemish, 2008; Schomerus *et al*., 2016; Ross *et al*., 2019).

UK newspaper coverage of mental illness has been found to be more frequently stigmatising (Murphy *et al*., 2013; Thornicroft *et al*., 2013; Rhydderch *et al*., 2016), portraying people with mental illness as hopeless victims or as perpetrators of violence and crime while neglecting discussion of treatment, recovery and personal experiences. Analyses from other European countries show similar patterns (Nawková *et al*., 2012; Aragonès *et al*., 2014; Ohlsson, 2018), finding that newspapers were likely to associate people with mental illness with stigmatising messages.

However, studies of UK and Canadian newspapers have shown improvement in coverage over the past decade (Whitley and Wang, 2017; Anderson *et al*., 2018). Both countries have long-running anti-stigma programmes that include work with media companies, which, along with the broader societal shift in perception of mental illness, have likely contributed these improvements (Henderson *et al*., 2016b; Whitley and Wang, 2017; Anderson *et al*., 2018).

In England, this programme is ‘Time to Change,’ delivered by the national charities Mind and Rethink, that includes social marketing (González-Sanguino *et al*., 2019), community-level projects and work with employers, schools and higher education institutions (Henderson and Thornicroft, 2009). Work with the media initially involved protesting stigmatising reporting, but now focusses on working with journalists, editors and writers, providing responsible reporting guidelines, workshops and a platform for discussion (Anderson *et al*., 2018).

The Time to Change responsible reporting guidelines (https://www.time-to-change.org.uk/media-centre/responsible-reporting) directly address eating disorders, self-harm and suicide. Other diagnoses are not discussed in detail. Previous analyses indicate that eating disorder stigma can be constructed differently by the media to that of other mental illnesses, but is still harmful (O'Hara and Smith, 2007; Shepherd and Seale, 2010; MacLean *et al*., 2015). Prior studies suggest that there is generally more stigma towards eating disorders than those with depression (Roehrig and McLean, 2010; Ebneter and Latner, 2013). This raises the question of whether coverage of eating disorders specifically has improved over time and how this coverage relative to coverage of other disorders has changed over time.

Other evidence suggests that the same question regarding coverage of schizophrenia should be examined, i.e. whether an overall improvement in coverage also applies to this diagnosis.

Surveys in several countries show that schizophrenia was associated with more stigmatising views than depression or bipolar disorder and attitudes have either not improved or worsened (Reavley and Jorm, 2012; Schomerus *et al*., 2012; Angermeyer *et al*., 2014, 2017). Previous analyses of UK newspaper coverage during the Time to Change programme also focussed on mental illness as a single construct (Thornicroft *et al*., 2013; Rhydderch *et al*., 2016; Anderson *et al*., 2018). However exploratory analysis (2018) indicated that a higher proportion of stories about schizophrenia were stigmatising than those about other diagnoses, in line with other studies of social media (Bowen and Lovell, 2019; Li *et al*., 2020) and newspapers (Goulden *et al*., 2011; Gwarjanski and Parrott, 2018; Bowen *et al*., 2019; Ross *et al*., 2019).

This study examines longitudinal trends of mental health coverage in the British press since the 2008 baseline for the whole of Time to Change, and since the 2016 baseline for its third phase. In addition, we compare coverage of each of eating disorders, schizophrenia and depression with coverage of all other disorders and examine for changes over time in these comparisons. Depression is a frequently covered condition allowing comparison with schizophrenia and eating disorders. Our hypotheses build on previous iterations of this study (Thornicroft *et al*., 2013; Rhydderch *et al*., 2016; Anderson *et al*., 2018): that there will be an increase in the odds that articles are anti-stigmatising, with a decrease in the odds that articles are stigmatising when comparing the findings from 2019 to 2008 and comparing 2019 to the findings from 2016. We will examine the variation in different stigmatising and anti-stigmatising themes reported on over the period from 2008 to 2019. We hypothesise that the odds that articles discussing depression were stigmatising would be lower than articles discussing other diagnoses; the odds that articles discussing schizophrenia or eating disorders were stigmatising would be higher than articles discussing other diagnoses and the trends relating to these three diagnoses would interact with the year an article was published.

## Method

This study utilises data previously collected relating to newspaper articles published from 2008 to 2016 (Thornicroft *et al*., 2013; Rhydderch *et al*., 2016; Anderson *et al*., 2018). The data for 2019 was collected using the same methods used in previous data collection rounds, to allow for direct comparison between 2019 and the previous years.

### Search strategy

The Lexis Nexis Professional UK electronic newspaper database was used to search articles from 18 local and national newspapers on two randomly chosen days each month which referred to mental illness. We ensured that there was a proportional representation of weekdays and weekend reports were included in the study, as per the data collection protocol used for previous data collection rounds. Ten national mass-circulation (>1 00 000 copies/day), daily newspapers and the eight highest circulation regional newspapers in England at the start of Time to Change were used. Only one newspaper per town/city was used to ensure geographical diversity. The Sun on Sunday is used from 2011 onwards to replace ‘News of the World’ which went out of print in July 2011. Only print news articles were included in the sample to allow for comparison between the different data collection rounds.

The following newspapers were included: Daily/Sunday Telegraph, Daily/Sunday Mail, Daily/Sunday Star, Daily/Sunday Express, Daily/Sunday Mirror, Times/Sunday Times, Sun/Sun on Sunday, Guardian/Observer, Independent/Independent on Sunday, Birmingham Evening mail, Eastern Daily Press (Norwich), Evening Chronicle (Newcastle), The Evening Standard (London), Hull Daily Mail, Leicester Mercury, Liverpool Echo, Manchester Evening News and The Sentinel (Stoke).

Newspaper articles from 2019 were retrieved on 24 randomly selected days using the Lexis Nexis database (Anderson *et al*., 2018). The search strategy included 35 general and diagnostic terms covering a wide range of mental disorders and descriptions of mental health services (Wahl, 1992): The full text of articles in the selected newspapers were searched using the following terms (* = wildcard): ‘mental health OR mental illness OR mentally ill OR mental disorder OR mental patient OR mental problem OR (depression NOT W/1 economic OR great) OR depressed OR depressive OR schizo! OR psychosis OR psychotic OR eating disorder OR anorexi! OR bulimi! OR personality disorder OR dissociative disorder OR anxiety disorder OR anxiety attack OR panic disorder OR panic attack OR obsessive compulsive disorder OR OCD OR post-traumatic stress OR PTSD OR social phobia OR agoraphobi! OR bipolar OR ADHD OR attention deficit OR psychiatr! OR mental hospital OR mental asylum OR mental home OR secure hospital’.

### Inclusion and exclusion criteria

Only articles that referred to clinical mental illness were included in the sample analysed, even if the reference was brief. Articles were excluded if they referenced a search term in a context unrelated to mental health (e.g. ‘the economy is depressed’); used in a non-clinical way (e.g. ‘Brexit is making me anxious’), or if a diagnostic or slang term was used metaphorically (e.g. ‘he is driving me nuts’). Articles relating primarily to developmental disorders (e.g. autism), neurodegenerative disorders (e.g. Alzheimer's disease), or substance use disorders alone were excluded as these were not the focus of Time to Change. Only articles published in the UK were included.

There was an increase in articles meeting inclusion criteria from 2016. A random sample of 50% of the articles for each day sampled in 2016 was coded and 67% of the articles for each day sampled in 2019 were coded. This ensured a similar sample size as for previous years and hence a manageable workload for coders.

### Coding

Articles were coded using content analysis (Krippendorff, 1989). Articles were first given a unique identifier derived from the date they were published, then coded for the newspaper of origin, diagnoses mentioned and the elements present in the article. An article may contain more than one type of diagnosis, or it may not contain any. If more than one diagnosis was present and discussed in different ways, the article was only coded for the dominant disorder. In response to the introduction of DSM-5, we included binge-eating disorder in the ‘eating disorders’ diagnostic category, which had previously been excluded.

Each article was read and analysed for the presence of specific elements, using the same coding criteria for previous work (Thornicroft *et al*., 2013; Rhydderch *et al*., 2016; Anderson *et al*., 2018). The elements describe the primary and/or secondary message conveyed by the article. Elements were derived from a combination of existing literature about mental health stigma and reporting, and the process of inductive coding (Thornicroft *et al*., 2013). Articles were also given a summary ‘overall’ code as stigmatising, anti-stigmatising, mixed or neutral. If an article contained stigmatising and anti-stigmatising elements that were given equal weight, the article was coded as mixed. If an article met the inclusion criteria, but none of the elements was present, the article was coded as neutral. If an article had a stigmatising element present, but this was overshadowed by anti-stigmatising elements, the article was only coded with the anti-stigmatising elements. Table 1 details the elements included in the analysis, and further details, including the coding framework, can be found in the online supplementary documents.

**Table 1.** Elements or central themes and ideas included in the article

Elements	
Stigmatising	Anti-stigmatising
1.1 Danger to others	2.1 Sympathetic portrayal of a person with mental illness
1.2 Problem for others	2.2 Causes of mental illness
1.3 Hopeless victim	2.3 Recovery from or successful treatment of mental illness
1.4 Strange behaviour	2.4 Mental health promotion
1.5 Personal responsibility causes	2.5 Mental health stigma
1.6 Sceptical of seriousness	2.6 Injustice
1.7 Pejorative/inappropriate language used	2.7 Prevalence of mental illness

The researcher coding the articles published in 2019 sampled for this iteration of the study was trained in the same way as those who coded previous years, other than the codebook developer (Thornicroft *et al*., 2013; Rhydderch *et al*., 2016; Anderson *et al*., 2018). All researchers were trained using articles from 2008 coded by the codebook developer and then coded another sample to derive the kappa value. The researcher coding the 2019 sample (RH) coded a sample of 92 articles from 2014 to derive the kappa value; this allowed him/her to discuss his/her results with the previous coder (CA) who had used the same sample. The agreement between coders was analysed using a *κ* analysis (Kirkwood and Sterne, 2003), and when a score higher than 0.7, indicating substantial agreement, was obtained, the coder was considered trained. Areas of discrepancy or uncertainty were discussed with CH and previous researchers until a consensus was reached.

### Analysis

First, the proportions of articles containing the various elements, diagnoses and overall category (stigmatising, anti-stigmatising, neutral or mixed) were calculated and compared. Univariate logistic regression models were used to estimate the odds that an article was stigmatising, anti-stigmatising, neutral or mixed in 2019 compared to 2008 and 2016 and the odds that an element would occur in 2019 compared to 2008 and 2016. Assumptions for the logistic regression models were checked for multicollinearity and linearity of the independent variables with the log odds was confirmed. Goodness of fit, outliers and appropriateness of the link function were checked using the deviance residuals. A Wald (*χ*^2^) test was used to assess the overall statistical significance of the year variable as a predictor in each model.

Three logistic regression models were constructed, one for each diagnosis, to compare the odds that an article was stigmatising if the diagnosis was present, adjusted for the year published and accounting for the hypothesised interaction. A Wald (*χ*^2^) test was used to assess the significance of the interaction between diagnosis and year published. Articles that did not discuss any named diagnosis were dropped from this part of the analysis.

Holm-Bonferroni sequential adjustments were used to account for 42 hypothesis tests: 14 individual elements plus four overall categories for the comparisons between 2008 and 2019 and 2016 and 2019; three tests assessing the association between stigmatising coverage and diagnosis and three tests describing interaction with year. The unadjusted level of statistical significance (*α*) was set as *p* = 0.05. All analyses were carried out using Stata version 16.0.

## Results

### The sample

The sample included 6731 articles, with: 880 from 2008, 794 from 2009, 626 from 1010, 694 from 2011, 1043 from 2013, 941 from 2014, 869 from 2019 and 880 from 2019.

### Changes in stigmatising and anti-stigmatising coverage

The frequencies and proportions of elements and overall categories by year are shown in Table 2. Stigmatising articles accounted for 46% of the coded articles published in 2008, 35% in 2016 and 23% in 2019. Anti-stigmatising articles accounted for 31% of the coded articles published in 2008, 50% in 2016 and 59% in 2019.

**Table 2.** Frequencies and proportions of elements and overall categories across articles, by year.

	2008^a^ (*n* = 882)		2009^a^ (*n* = 794)		2010^a^ (*n* = 629)		2011^a^ (*n* = 694)		2013^a^ (*n* = 1043)		2014^a^ (*n* = 941)		2016^a^ (*n* = 869)		2019 (*n* = 880)		Total (*n* = 6731)	
	*n*	%	*n*	%	*n*	%	*n*	%	*n*	%	*n*	%	*n*	%	*n*	%	*n*	%
Overall category																		
Neutral	144	16	120	15	69	11	57	8	238	23	132	14	82	9	126	14	968	14
Stigmatising	406	46	340	43	315	50	315	45	178	17	411	44	300	35	202	23	2467	37
Anti-stigmatising	273	31	284	36	211	34	285	41	366	35	331	35	437	50	515	59	2704	40
Mixed	58	7	48	6	29	5	37	5	261	25	67	7	50	6	37	4	587	9
Elements – stigmatising																		
Danger to others	186	16	138	14	129	20	95	10	74	5	109	9	149	13	93	8	973	11
Problem for others	62	5	85	9	54	8	50	5	38	2	64	5	54	5	63	6	470	5
Hopeless victim	137	12	72	7	83	13	152	16	115	7	275	22	123	10	50	5	1007	11
Strange behaviour	108	9	72	7	58	9	93	10	206	13	152	12	89	7	50	5	828	9
Personal responsibility	114	10	52	5	20	3	11	1	2	0	77	6	41	3	35	3	352	4
Sceptical of seriousness	18	2	21	2	6	1	19	2	53	3	29	2	26	2	11	1	183	2
Pejorative language	49	4	61	6	26	4	31	3	111	7	50	4	31	3	23	2	382	4
Elements – anti-stigmatising																		
Sympathetic portrayal	202	17	193	20	70	11	141	15	168	10	117	9	268	23	231	21	1390	16
Causes of mental illness	117	10	127	13	68	11	78	8	355	22	83	7	139	12	186	17	1153	13
Recovery/treatment of mental illness	76	6	53	5	60	9	97	10	223	14	95	8	49	4	130	12	783	9
Mental health promotion	59	5	41	4	26	4	125	13	80	5	107	8	115	10	123	11	676	8
Stigma	11	1	16	2	7	1	16	2	56	3	34	3	38	3	33	3	211	2
Injustice	11	1	16	2	7	1	16	2	56	3	34	3	38	3	33	3	211	2
Prevalence	23	2	25	3	25	4	27	3	65	4	38	3	28	2	46	4	277	3

a Data reported for 2008, 2009, 2010, 2011, 2013, 2014 and 2016 were collected in previous iterations of the study, using the same data collection protocols. The previous study iterations are reported in Thornicroft *et al*. (2013), Rhydderch *et al*. (2016) and Anderson *et al*. (2018).

The results of the logistic regression models relating to changes in stigmatising and anti-stigmatising coverage over time are presented in Table 3. In support of the hypotheses that there was an increase in anti-stigmatising content and decrease in stigmatising content, the odds that an article was anti-stigmatising was 3.16 times higher in 2019 compared to 2008 (OR 3.16 (2.60–3.84), *p* < 0.001). There was a 40% increase in the odds that an article was anti-stigmatising between 2016 and 2019 (OR 1.40 (1.16–1.69), *p* < 0.001). Between 2008 and 2019, the odds that an article was stigmatising reduced significantly (OR 0.35 (0.28–0.43) *p* < 0.001). The odds that an article was stigmatising reduced significantly between 2016 and 2019 (0.56 (0.46–0.70), *p* < 0.001). In all cases, the Wald (*χ*^2^) tests (reported in Table 3) were positive for the overall statistical significance of the year variable as the predictor in each model.

**Table 3.** Results from the logistic regression models comparing the association between the odds that a stigmatising element or anti-stigmatising element is present in 2019 compared to (a) 2008 and (b) 2016

Element	(a) Odds ratio (95% CI) 2008–2019	*p*-value	(b) Odds ratio (95% CI) 2016–2019	*p*-Value	*χ*^2^(7) test statistic ^a^	*p*-value
Stigmatising elements						
Danger to others	*0.44 (0.34–0.58)	<0.001	*0.57 (0.43–0.75)	<0.001	102.14	<0.001
Problem for others	1.02 (0.71–1.46)	0.926	1.16 (0.80–1.69)	0.437	36.69	<0.001
Hopeless victim	*0.33 (0.23–0.46)	<0.001	*0.36 (0.26–0.51)	<0.001	249.26	<0.001
Strange behaviour	*0.43 (0.30–0.61)	<0.001	*0.53 (0.37–0.75)	<0.001	113.14	<0.001
Personal responsibility causes	*0.28 (0.19–0.41)	<0.001	0.83 (0.53–1.32)	0.442	133.00	<0.001
Sceptical of seriousness	0.61 (0.28–1.29)	0.194	0.41 (0.20–0.83)	0.014	35.17	<0.001
Pejorative language	*0.46 (0.27–0.78)	0.002	0.72 (0.42–1.25)	0.247	75.67	<0.001
Anti-stigmatising elements						
Sympathetic portrayal	1.19 (0.96–1.48)	0.109	0.80 (0.65–0.98)	0.031	160.89	<0.001
Causes of MI	*1.75 (1.36–2.25)	<0.001	1.40 (1.10–1.79)	0.006	285.90	<0.001
Recovery from MI	*1.83 (1.36–2.47)	<0.001	*2.89 (2.05–4.07)	<0.001	158.63	<0.001
MH promotion	*2.26 (1.63–3.13)	<0.001	1.06 (0.81–1.39)	0.664	127.31	<0.001
Stigma	*3.08 (1.55–6.13)	0.001	0.85 (0.53–1.37)	0.503	42.24	<0.001
Injustice	*3.30 (2.30–4.75)	<0.001	*1.62 (1.21–2.19)	0.001	99.13	<0.001
Prevalence	2.06 (1.23–3.42)	0.006	1.65 (1.02–2.67)	0.040	22.66	0.002
Overall element						
Neutral	0.85 (0.65–1.09)	0.21	*1.60 (1.19–2.15)	0.002	104.11	<0.001
Stigmatising	*0.35 (0.28–0.43)	<0.001	*0.56 (0.46–0.70)	<0.001	363.18	<0.001
Anti-stigmatising	*3.16 (2.60–3.84)	<0.001	*1.40 (1.16–1.69)	<0.001	224.83	<0.001
Mixed	0.622 (0.41–0.95)	0.028	0.72 (0.46–1.11)	0.135	350.35	<0.001

a Wald test assessing the significance of the year variable in the model, with 7 degrees of freedom.

*Odds ratio is statistically significant at the 5% level after Holm Bonferroni adjustment.

There was a significant increase in the anti-stigmatising elements ‘recovery/successful treatment of mental illness’ (OR 2.89 (2.05–4.07), *p* < 0.001,) and ‘injustice’ (OR 1.62 (1.21–2.19), *p* = 0.001) between 2016 and 2019, and a statistically significant increase in all anti-stigmatising elements except for ‘sympathetic portrayal’ and ‘prevalence’ between 2008 and 2019, shown in Table 3. There was a statistically significant decrease in all stigmatising elements except for ‘sceptical of seriousness’ and ‘problem for others’ between 2008 and 2019. There was a significant decrease in the stigmatising elements ‘danger to others,’ (OR 0.57 (0.43–0.75), *p* < 0.001) ‘hopeless victim,’ (OR 0.36 (0.26–0.51) *p* < 0.001) and ‘strange behaviour’ (0.53 (0.37–0.75), *p* < 0.001) between 2016 and 2019.

### Diagnosis, stigmatising coverage and changes over time

As shown in Table 4, depression was the most common diagnosis and was discussed in 31% of articles in the sample; with 3% of articles in the sample discussing schizophrenia and 7% discussed eating disorders. Of the total, 32% did not specify a diagnosis, so were removed from further analysis, leaving 5142 articles.

**Table 4.** Frequencies and proportions of diagnoses across articles, by year

Diagnosis	2008 (*n* = 501)		2009 (*n* = 475)		2010 (*n* = 444)		2011 (*n* = 340)		2013 (*n* = 758)		2014 (*n* = 623)		2016 (*n* = 550)		2019 (*n* = 550)		Total^a^ (*n* = 4241)^b^	
	*n*	%	*n*	%	*n*	%	*n*	%	*n*	%	*n*	%	*n*	%	*n*	%	*n*	%
Depression	267	28	244	29	260	38	207	24	370	31	352	33	342	34	299	29	2341	31
Bipolar	29	3	57	7	25	4	43	5	61	5	44	4	51	5	12	1	322	4
Schizophrenia	50	5	41	5	62	9	55	6	58	5	41	4	71	7	32	3	410	5
Eating disorder ^c^	78	8	72	8	55	8	55	6	90	8	66	6	64	6	46	5	526	7
Anxiety disorder	34	4	40	5	25	4	27	3	46	4	53	5	43	4	112	11	380	5
PTSD	36	4	29	3	26	4	46	5	44	4	69	7	47	5	98	10	395	5
OCD	8	1	10	1	8	1	9	1	27	2	13	1	15	1	23	2	113	1
Personality disorder	10	1	16	2	18	3	14	2	0	0	14	1	7	1	10	1	89	1
Agorophobia	0	0	0	0	0	0	9	1	4	0	6	1	3	0	4	0	26	0
Postnatal depression	16	2	10	1	0	0	16	2	22	2	14	1	21	2	12	1	111	1
ADHD	28	3	16	2	18	3	17	2	23	2	27	3	23	2	25	2	177	2
Other disorder	3	0	0	0	4	1	26	3	154	13	41	4	9	1	15	1	252	3

a Total number of times the diagnosis is mentioned.

b Number of articles included in the analysis.

c “Eating disorder category includes but is not limited to Bulimia nervosa and Anorexia nervosa.

*Note*: Data reported for 2008, 2009, 2010, 2011, 2013, 2014 and 2016 were collected in previous iterations of the study, using the same data collection protocols. The previous study iterations are reported in Thornicroft *et al*. (2013), Rhydderch *et al*. (2016) and Anderson *et al*. (2018)

**Fig. 1.**
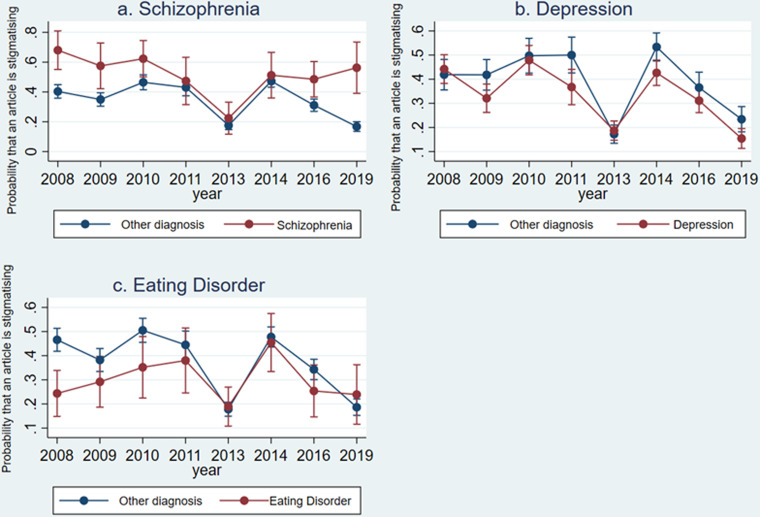
Results from the predictive marginal models showing the probability that an article is stigmatising if the article discusses (*a*) schizophrenia, (*b*) depression or (*c*) eating disorders compared to other diagnoses, with 95% confidence intervals.

Articles about schizophrenia were 6.37 times more likely to be stigmatising than articles discussing any other diagnosis (OR: 6.37 (3.05–13.29) *p* < 0.001) and a Wald test indicated that there was a significant (*p* = 0.01) interaction between the year an article was published and the odds that an article about schizophrenia was stigmatising. The results of the regression analysis are shown in Table 5 and Fig. 1*a* shows that in 2008, articles about schizophrenia were more likely to be stigmatising than those that were not, but this discrepancy became insignificant between 2010 and 2014. In 2016 and 2019, the probability that a stigmatising article was about schizophrenia remained comparable to the probability a stigmatising article was about schizophrenia in 2008 and 2009. Over the same period, the probability that articles about other diagnoses were stigmatising dropped significantly.

**Table 5.** Results from the logistic regression models (a) Odds ratio describing the odds that an article is stigmatising when the diagnosis is present *v.* the diagnosis not being present, adjusted for the year published and (b) Wald test showing the significance of the interaction between a diagnosis being associated with being stigmatising and the year published

Diagnosis	(a)OR** (95% CI)	*p*-value	(b) *χ*^2^(7) test statistic^a^	*p*-value
Schizophrenia	6.37 (3.05–13.29)	*<0.001	18.42	0.010*
Depression	0.59 (0.69–0.85)	*0.018	13.11	0.070
Eating disorder^b^	1.37 (0.97–2.80)	0.870	12.73	0.080

a Wald test assessing the significance of the year variable in the model, with 7 degrees of freedom.

b Eating disorder category includes but is not limited to Bulimia nervosa and Anorexia nervosa.

*Significant with 95% confidence after Holm Bonferroni adjustment.

** Odds ratio indicating that the diagnosis is associated with an article being stigmatising (baseline: neutral/anti-stigmatising/mixed).

Articles about depression were significantly less likely to be stigmatising than articles about other diagnoses (OR 0.59 (0.69–0.85) *p* = 0.018). The Wald test indicated that the interaction between the year an article was published and the odds that an article about depression was stigmatising was not statistically significant (*p* = 0.07). Figure 1*b* shows that the pattern of change over the study period was similar for both the depression group and the ‘other diagnosis’ group. These results are shown in Table 5.

There was no evidence for a difference in stigmatising coverage of eating disorders *v.* other diagnoses (OR 1.37 (0.67–2.80) *p* = 0.386). The Wald test indicated that the interaction between the year variable and stigmatising coverage of eating disorders was not statistically significant (*p* = 0.08). Figure 1*c* shows the pattern of this change and while eating disorders were discussed in a less stigmatising way than other diagnoses in 2008, this gap closed as coverage of other diagnoses improved.

## Discussion

The study provides the first evidence of a sustained improvement in the discourse around mental illness in print media, following initial findings of improvement by Anderson *et al*. (2018). The number of articles retrieved for 2019 was higher than most previous years, except for 2016, supporting previous findings that coverage of stories relating to mental illness is generally increasing (Murphy *et al*., 2013; Anderson *et al*., 2018). Thus, there is an increase and an improvement in reporting about mental illness, with a reduction in the proportion of stigmatising articles and an approximately proportional increase in anti-stigmatising articles.

Improvements in knowledge about and attitudes towards mental illness showed improvements since 2014 (Henderson *et al*., 2016a) and with the continuation of improvement in 2017 (Robinson and Henderson, 2019) and 2019 (Henderson *et al*., 2020). A similar pattern, albeit delayed, is seen within newspaper reporting: coverage between 2008 and 2014 showed no significant reduction in the proportion of stigmatising coverage (Thornicroft *et al*., 2013; Rhydderch *et al*., 2016), followed by a significant reduction in stigmatising coverage in 2016 (Anderson *et al*., 2018) that was sustained in 2019. There was a reduction in the proportion of stigmatising articles in 2013, but this change was not sustained and was not associated with an increase in anti-stigmatising articles.

While it has been previously shown that stigmatising articles effect population attitudes towards mental illness (Thornton and Wahl, 1996; Corrigan *et al*., 2005, 2013; Klin and Lemish, 2008; Schomerus *et al*., 2016; Ross *et al*., 2019), it is possible that the causal pathway is not unidirectional. The public may have had more access to positive stories about mental illness via the internet, often relating to recovery or treatment of mental illness, which may then affect their perceptions and the views of traditional journalists (Betton *et al*., 2015; Carmichael *et al*., 2019; González-Sanguino *et al*., 2019).

The increase in stories discussing recovery from mental illness is particularly encouraging. Research suggests that stories portraying individuals constructively coping with mental illness can benefit others who are similarly struggling (Niederkrotenthaler and Till, 2019; Til 2019). However, while social media and web-based forums can reach hard-to-engage populations, the lack of accountability in social media can allow the spread of misinformation about mental illness and cause harm (for example, through cyberbullying), to vulnerable people (Daine *et al*., 2013; Robinson *et al*., 2016).

The finding that schizophrenia is associated with more stigmatising newspaper coverage is in line with other studies (Clement and Foster, 2008; Bowen *et al*., 2019; Ross *et al*., 2019). This study shows that the proportion of stigmatising articles about schizophrenia has recently increased. The disproportionate proportion of stigmatising coverage associated with schizophrenia could be for several reasons. Schizophrenia is frequently associated with violence and criminality when discussed in newspapers (Clement and Foster, 2008; Goulden *et al*., 2011; Aoki *et al*., 2016; Rodrigues-Silva *et al*., 2017; Gwarjanski and Parrott, 2018; Bowen *et al*., 2019), either in a metaphorical or literal sense. Newspapers focus on criminality and mission to report topics that are ‘newsworthy’ may create a selection bias towards only publishing stories about people with schizophrenia that have committed a criminal act. However, reports of criminal behaviour can discuss the role of an individual's mental disorder in a neutral or anti-stigmatising way. Population prevalence of psychotic disorders is much lower than that of depression (McManus S *et al*., 2016). As not knowing someone with a mental disorder is associated with more stigmatising views (Henderson *et al*., 2020), there may not be the same demand for sensitive, anti-stigmatising reports of schizophrenia in the way that there is for other disorders and people with schizophrenia may be less likely to be asked to contribute their experiences to stories due to this lower prevalence and unchallenged prejudice.

This study showed that articles discussing depression were consistently less likely to be stigmatising than other articles, consistent with findings that population attitudes to depression tend to be less stigmatising than those towards other disorders (Reavley and Jorm, 2012; Schomerus *et al*., 2012; Angermeyer *et al*., 2014).

While eating disorders are much less common than depression and other common mental disorders (Micali *et al*., 2013; McManus *et al*., 2016), the reporting of eating disorders was found to be no more or less stigmatising than that regarding other disorders. It is discussed more than all other diagnoses apart from depression, indicating that despite the relative rarity of eating disorders, they are widely discussed. The reporting guidelines provided for reporters by Time to Change and other mental health charities may have also improved the quality of coverage relating to eating disorders, making them no less stigmatised than other disorders.

### Strengths and limitations

A major strength of this study is that it is an ongoing longitudinal dataset, which is a detailed and consistent analysis of newspaper coverage of mental illness for over a decade. While adhering to the protocol developed for the initial round of data collection has limited the scope of this study (i.e. the exclusion of online news sources and exclusion of certain diagnoses), this consistency has allowed for an in-depth understanding of the way that portrayals of mental illness have changed during the period. However, while newspapers still play a significant role in shaping national attitudes towards mental illness, this influence has declined since this study started in 2008 as more people use social media as a source of news and information.

Headlines and photographs were not analysed in this study. The exclusion of photographs may have disproportionately reduced sensitivity of the study in identifying eating disorder stigma in comparison to other mental illnesses (Bowen *et al*., 2020). The coding framework was, however, carefully designed, referencing a wide range of sources and the use of inductive coding to assess stigma in a wide range of mental illnesses, including eating disorders.

The analysis of changes in stigma associated with articles discussing schizophrenia was a novel addition to this study. However, further insight into the details of, for example, the variation in the stigmatising or anti-stigmatising elements was not possible in this dataset, as the sample did not have the power to support such a granular analysis. Further, we could not examine changes over time for all diagnoses due to low frequencies within the dataset.

The decision by the study to exclude articles relating to neurodegenerative, neurodevelopmental and substance use disorders further limits the scope of the study, although this omission was integral to the overall aim of the study to assess the impact of the Time to Change programme on the stigma associated with mental illness in UK media.

### Implications for anti-stigma programmes

Our findings suggest that the work by Time to Change is associated with a reduction in the proportion of stigmatising newspaper articles about mental illness in the UK. However, a wide range of factors may have contributed to this change, interventions such as Time to Change must continue to work with journalists and the media, although the focus could be updated.

Specific guidelines about reporting on schizophrenia should be developed, as for those on eating disorders (Angermeyer and Matschinger, 2003). That the difference in frequency of stigmatising reports relating to schizophrenia and those about other disorders widening is cause for concern. As has been observed with eating disorder stigma, the stigma associated with schizophrenia can have different features to that related to depression, and this will need to be accounted for in future work by Time for Change (Angermeyer and Matschinger, 2003). To gain further insight, future evaluations of Time to Change could include outcomes relating to specific mental illnesses. Current interventions may not be helping all people with mental illness equally, so it will be essential to assess knowledge, attitudes and behaviours relating to different diagnoses.

## Supplementary material

For supplementary material accompanying this paper visit https://doi.org/10.1017/S204579602000089X. click here to view supplementary material

## Availability of data and materials

The dataset of newspaper articles and the coding dataset are available upon reasonable request.
