# Supplementary material for: Improvement for most, but not all: changes in newspaper coverage of mental illness from 2008 to 2019 in England
Source: Epidemiol Psychiatr Sci. 2020 Nov 5;29:e177. doi: 10.1017/S204579602000089X (PMC7681112; doi:10.1017/S204579602000089X)
Supplement: Supplementary file 1 [file S204579602000089Xsup001.docx]

Improvement for most, but not all: Changes in Newspaper Coverage of mental Illness from 2008 to 2019 in England: Supplementary Material

# Search Strategy

The full text of articles were searched using the following terms (!=wildcard): ‘mental health OR mental illness OR mentally ill OR mental disorder OR mental patient OR mental problem OR (depression NOT W/1 economic OR great) OR depressed OR depressive OR schizo! OR psychosis OR psychotic OR eating disorder OR anorexi! OR bulimi! OR personality disorder OR dissociative disorder OR anxiety disorder OR anxiety attack OR panic disorder OR panic attack OR obsessive compulsive disorder OR OCD OR post-traumatic stress OR PTSD OR social phobia OR agoraphobi! OR bipolar OR ADHD OR attention deficit OR psychiatr! OR mental hospital OR mental asylum OR mental home OR secure hospital’.

# Inclusion and Exclusion Criteria

## Include

- - Articles which talk about clinical mental illness, no matter how brief the reference.
  - There need not be a specific reference to clinical services, merely a description of an illness which likely meets clinical level.
  - The article can be about mental health (MH) services or organisations, but if it’s about the latter in particular, make sure it’s talking about clinical mental illness; MH charities often do work about ‘wellbeing’ or mild problems, which are not being analysed.
  - If it is unclear if something being discussed is clinical or not (e.g. about depression) think about how severe, chronic the symptoms being described sound. If a phrase like ‘suffering from depression’ is used, that would suggest clinical. If potentially nonclinical terms like ‘anxiety’ and ‘depression’ are used along with a general term such as ‘mental health’, generally code unless there is specific evidence that it is trivial.
  - Suicide and self-harm are not searched for. If, however, a story comes up featuring it, then include, as this means that either a diagnosis or a general term (including psychiatr*) has bought it up, thus making the link with MI.
  - If it’s speculation about MI, include if the speculation seems to have some plausibility: this is the often the case where somebody convicted of a crime is sent for psychiatric evaluation. If, however, a psychiatric problem has been explicitly rejected, then exclude.

## Exclude

- - If the use is nonclinical
    - ‘Stress’ at work, especially in the absence of a term like ‘mental health’. However if a term like mental health is used, and it talks about long-term sick leave, for example, then include.
    - If it is talking about short term symptoms: ‘millions are likely to phone in sick today on what psychologists call the most depressing day of the year’ then exclude.
    - ‘Depressed’ is particularly likely to be used non-clinically, especially when it’s with ‘feeling depressed’: again, judge based on context, chronicity etc. Often ‘depressed’ alone is not enough: either needs more specific indicator that it’s a clinical problem, or a qualifier such as ‘chronically depressed’, or a term like ‘a depressive’. If it’s described as an ‘illness’ or listed with other illnesses (inc. anxiety), then include it; if it’s listed in a list with other nonclinical symptoms e.g. ‘an increase in stress, loneliness, depression…’, then likely exclude.
    - In problem pages, depression is often speculated on in the agony aunt’s reply; if the symptoms sound severe, include, but if it is less so (general relationship troubles) then less likely.
    - A few incidences of depression being described as a symptom/side-effect of physical illness, medication or quitting smoking; generally exclude as this is usually short term However, if it’s clearly serious and talks about it leading to a longer term problem, then include. Stories about smoking leading to a mental illness (and not just short-term symptom) *are* included.
    - An unspecific reference to ‘mental health’ or ‘effects on mental health’, with little indication that the article is talking about serious symptoms, would be excluded.
    - References to ‘mental health’ in the same context as other physical illnesses would be included.
    - If the article is about wellbeing and ‘positive’ mental health stories; only include when it’s suggested that it is about preventing clinical mental health problems.
    - If the article refers to MH services/service providers without talking about their work, exclude.
    - If the use is trivial e.g. ‘This weather’s making me depressed’.
    - If it is about the following disorders:
      - Dementia.
      - Autism.
      - Paedophilia or paraphilia.
      - Learning difficulties.
      - However, if an article about one of these disorders also involves comment/discussion about MI more generally, then include it.
      - Substance abuse also excluded, except if the article is about that causing MH problems (as opposed to it as an MH problem itself).
      - A medical situation in which a psychiatrist is involved but it is not for a mental disorder e.g. hypnotherapy, some non-clinical personality issue. Any non-specific mental problem requiring psychiatric care is, however, included.
      - If it is people receiving short-term psychiatric care in response to trauma e.g. crime, unless there’s indication that the problem develops into something long term (e.g. depression or PTSD). However, even if long term, the chaotic nature of the victim’s life might be such that it would be hopeless victim, given the exceptional circumstances make empathy difficult.
      - If it is a non-specific psychiatric problem resulting from brain damage i.e. what seem to be neurological problems.
    - If the use is metaphorical e.g. ‘His behaviour is schizophrenic’.
    - If the use is incidental: ‘John Smith, the son of an investment banker and psychiatrist, has decided to…’
      - This might be unclear: sometimes what might initially seem incidental is in fact deliberate/considered and intended to convey a meaning. Only exclude if it is clear the use is entirely incidental then exclude.
    - If the article is about animals’ MH.

# Coding framework for stigmatising and anti-stigmatising elements

The coder should ask themselves if the article stigmatizing, anti-stigmatizing, neither, or both? What are the elements that make it one or the other? How prominent are the elements in the article?

Articles can contain numerous elements, but generally some have more weight than others. The aim here is not to meticulously code what *ideas are mentioned*, but to code what *messages are conveyed*. In practice this involves looking at what elements are contained in the article, considering how they are conveyed and how much weight they are given, and thus forming an overall impression. Whatever you decide is the overall tone of the article, do not code contradictory elements: only when you decide the article is genuinely mixed, code for both stigmatizing and anti-stigmatizing elements; these must be on the same level (i.e. both primary or both secondary; if one is greater then that is the tone). When two elements are contained in the primary section, think about which comes first, is more prominent. The rationale for this is that most readers will form a simplified take home message from a piece. When no messages are conveyed, code as neutral.

## Elements

1. Stigmatizing
   1. Danger to others
      - *Meaning: people with a mental illness are dangerous.*
      - This might often, in fact, be a secondary category e.g. a story about a murder which only mentions the Mental Illness (MI) late on. Make sure to code this accurately.
      - This isn’t just ‘violence’: includes e.g. man with depression driving dangerously, man with MI indecently exposing himself.
      - Articles that describe a person with MI who has committed a violent act sensitively and/or sympathetically may not convey this meaning – don’t automatically code all articles about criminal behaviour committed by a person with MI as 1.1.
      - Need not specifically point to a causal relationship between MI and the danger; the association is enough e.g. talking about the MH problems of a groups widely considered dangerous such as prisoners.
        - However, do not code if it is *explicitly* stated that MI developed *after* the violent incident. In this case, code 1.3 hopeless victim and perhaps 1.5 personal responsibility cause.
      - Often stories in which prisoners or criminals’ MI is offered to show the injustice they are facing e.g. they should be better treated, shouldn’t be executed. This could make it ‘injustice’, but generally code as violent, as their status means that for most people they can’t really be sympathized with.
      - Co-coding with strange behaviour: ‘suicide by cop’ stories will often involve 1.1 and 1.4, as will be story in which non-violent, strange psychiatric symptoms are discussed in addition to the violence.
   2. Problem for others
      - *Meaning: people with a mental illness make problems for others for no good reason.*
      - Includes all forms of problem-making for others, which don’t come under ‘danger’.
      - Make sure it’s actually causing problems for others and isn’t in fact just strange behaviour; sometimes it’s both.
      - A sympathetic portrayal (2.1) might nonetheless suggest that someone with a MH problem is a problem for others (1.2). However, *only* code 1.2 when there is a suggestion of ‘problem for others’ without any sympathetic details (e.g. ‘His early life was marred by his depressive father’s behaviour, but he went on to….’) i.e. when the person with mental illness is referenced *exclusively* as a burden.
        - Includes more general references: ‘Mental illness, gangs and educational underachievement are just some of the ills that judges, teacher and politicians have blamed on “the breakdown of the family” in recent weeks’ with no other sympathetic info on MI.
   3. Hopeless victim
      - *Meaning: people with a mental illness are weak, helpless and pitiful, to an extent that you can not relate to them.*
      - See 2.6 for more on this.
      - Any depiction of someone with MI as tragic, pitiful, a failure, leading a life full of chaos etc.: like with the other categories, this need not be explicitly linked to the MI; just any negative depiction of someone with an MI where they are primarily the victim.
        - As is always the case, the mere mention of any of these things is not what’s coded, but a strong focus on them.
      - Differs from 1.2 in that the ‘victim’ of the problematic behaviour is the person themselves, not others.
      - Suicide stories often come under this (see above).
      - Look out for adjectives like ‘tragic’, and ‘troubled’.
      - Story more likely to meet this criteria when the misfortune suffered is serious (e.g. attacked, suicide); milder problems are less likely to imply ‘hopelessness’ or ‘tragedy’.
      - Include stories about prisoners who have an MI and face some kind of injustice because this is sympathetic and portrays them as a victim, but hardly as victims we sympathise with, or are ‘normal’; being in prison is a pretty good example of hopeless life details. Exceptions might be if they or their family are quoted a lot, in which case it might get a 2.6, but then this will often be ‘Mixed’ with danger or problem to others as they are convicted criminals.
      - Rare to code this along with more ‘active’ categories like ‘personal responsibility’.
   4. Strange behaviour
      - *Meaning: people with a mental illness are strange.*
      - Unusual behaviour (1.4) likely appears in many articles. If this is done sensitively and explored properly, don’t code for 1.4, but if it is sensationalist (i.e. features in the headline) or poorly explained, code for 1.2.
      - ‘Irrational’ (i.e. lacking a psychosocial stressor) self-harming behaviour or a suicidal act with bizarre circumstances. Less likely when the person has depression, as this is generally regarded as a response to some psychosocial stressor (even if not mentioned), but more likely when it’s non-specific ‘mental health problems’ or schizophrenia.
      - Instance whereby mental illness is used for ‘comic’ purposes.
        - However, as with all codes, even this can be done sensitively, esp. when done by a person with mental illness.
      - For eating disorders, lurid descriptions of people being extremely thin are coded 1.4.
      - The behaviour needn’t itself be directly linked to the MI, but when MI is talked about in the context of unusual behaviour, code. Thus this might often be a good code for an unsympathetic biography in which it is mentioned that they have an MI (as a secondary element): the person may be made to seem unsympathetic by their strange or unusual behaviour.
      - Troubled genius stories often come under here.
   5. Personal responsibility causes
      - *Meaning: people with a mental illness are to blame for their illness.*
      - This would typically involve implicating ‘problematic’ lifestyles which the person has *freely* chosen themselves e.g. drugs, alcoholism, extreme dieting, overworking.
        - If, however, the ‘choice’ is in a context which suggests they were under a lot of pressure and it was actually an understandable response, then don’t necessarily code. Again, it’s about emphasis etc.: if it features prominently, more likely to code.
        - Surveys suggest drug and alcohol consumption is overwhelmingly seen as a free choice: there would have to be good contextual explanation of this behaviour not to code it.
        - If the alcoholism is clearly a response to the illness (‘self-medicating’), perhaps not code, unless it’s implicated in the illness’s persistence.
      - There doesn’t have to be a direct causal claim e.g. ‘a depressive alcoholic’ is enough.
      - Could also include pieces which suggest that depression results from a character flaw.
      - Could also include piece in which people suffer psychosocial stress because they chose to put themselves through high stress experience e.g. adhering to unreasonable social norms, overworking.
      - Include pieces in which people’s continued suffering result from their own choice, perhaps through refusing to take medication.
      - Look out for terms like ‘self-destructive’.
      - Can also be good for stories which aren’t about MI so much, but an unsympathetic account of someone who turns out to have an MI; it may be that they have it due to their bad lifestyle choices etc. Includes prisoners, criminals etc.
   6. Sceptical of seriousness
      - *Meaning: mental illness is not a real illness; people with it just need to pull themselves together or are trying to get attention.*
      - Suggestions that people with MI just need to ‘pull themselves together’ or that they are faking it.
      - Sometimes fine line between sceptical of seriousness, personal responsibility, and non-clinical. Generally, if the diagnosis is clearly meant seriously, but the blame is personal or it’s a ‘choice’, then it’s 1.5. 1.6 is when a clearly clinical term is used, or is claimed by the sufferer, but somebody suggests it’s not that serious. For example, articles where people with anorexia and bulimia are described as ‘diet-obsessives’ could be coded as 1.6.
      - This does not include ‘Sceptical of authenticity’, in which it is *reasonably* suspected that someone is in fact consciously faking an MI for nefarious purposes. However, unless there’s good reason beyond the columnist casting doubt, then code it.
      - A well argued piece suggesting that a particular MI is over-diagnosed is not coded, provided it’s made clear that some people genuinely do suffer it. Rather, this is neutral, as not much about the ‘real’ MI is actually being said.
   7. Pejorative/inappropriate language
      - *Meaning: this use of language about people with a mental illness evokes prejudicial ideas or reduces them to their illness.*
      - Think about *meaning*: pejorative terms when used in certain contexts and by certain people don’t carry a pejorative meaning.
      - Pejorative language about MH services is also coded e.g. ‘shrink, ‘asylum’.
      - Any penal term used in relation to a psychiatric hospital e.g. incarcerated, cell, detained, released
      - Reductionist or totalising language e.g. ‘a schizophrenic’, ‘a mental patient’, saying someone ‘*is* mentally ill’, talking about ‘the mentally ill’. The term ‘schizophrenic killer’ would be stigmatizing because it associates MI and violence *and* because it uses totalising language (it could instead have said ‘killer who has schizophrenia’) hence code for both.
      - This will rarely be a primary element.
      - In a legal context, ‘insane’ is in fact an accepted term, so don’t code in these instances.
      - Terms like ‘troubled’ and ‘tragic’ don’t come here, but will often imply ‘Hopeless victim’.
2. Anti-stigmatizing
   1. Sympathetic portrayal
      1. General public
      2. Public figure
      - *Meaning: someone I like or can relate to can get a mental illness.*
      - This is the most basic type of coding which essentially says ‘anti-stigmatizing’ in the absence of something more specific; can be individual or defined group.
      - Do not code if the ‘group’ is simply People with Mental Illness (PWMI), and they are not given another identity (or at least a specific subset of (PWMI) e.g. in a particular hospital; in this case code it and put group: PWMI in notable).
      - If it’s a defined group (up to and including a whole profession), it comes here, but if it’s bigger than that (e.g. ‘women’, ‘children’) it may come under prevalence.
      - ‘Portrayal’ doesn’t mean a ‘life story’, but merely details, no matter how brief, about the life of any individual who has experience an MH problem that is portrayed sympathetically. This could be as little as ‘With Vaughan injured and Marcus Trescothick suffering from depression in 2005-06, Andrew Flintoff led England…’ This would obviously be a ‘secondary element’, but the point is that it refers to MI as something that ‘normal’ people have (but make sure it is non-pejorative, non-judgemental, doesn’t meet any of the grounds for exclusion noted below, and is not about someone who has been vilified). In this sense it can be more of a ‘Non-judgmental mention’ than a ‘Sympathetic portrayal’. However if the reference is very brief, and we get no information about the person (positive or negative) it may just be neutral.
      - Indications that a portrayal is not sympathetic (guidelines, not rules):

- The person is reduced to their mental illness, with little reference to their social identity (family, job etc.).
- Their bizarre behaviour is focused on.
- Pejorative language is used.
- The person is not allowed to speak for themselves.
- It is about a person(s) who is vilified in the piece or is generally not seen favourably e.g. criminal (this would often be 1.1. or 1.2).
  - - However, there are many instances of sympathetic portrayals – in which the individual themselves talk about their illness at length or their illness is discussed sensitively at length – that also has stigmatizing elements alongside. In this instance, code both the stigmatizing element *and* sympathetic portrayal as primary. However, you can’t have a primary stigmatizing element and a secondary sympathetic portrayal element. If the sympathetic portrayal is only secondary (i.e. a short reference), then the primary stigmatizing element ‘outweighs’ it, and it is not coded. The point of the secondary ‘brief reference’ (see Trescothick example above) is that it shows ‘normal’ people get MI; this clearly doesn’t apply in a context in which the person has already been stigmatized.
    - If it’s in a fictional work, there needs to be a bit more detail about the person than if it’s real life; does the character seem like one we would like or dislike? Also true if it’s real life but there’s really very little information about the person; however, the very fact that they’re a real person who openly has an MI might be enough, whereas with a fictional portrayal this doesn’t apply. If it’s unclear, code neutral.
    - If it is a relative of a public figure, code as ‘Public figure’.
    - A fictional portrayal by a famous actor is ‘General public’.
    - Public figure has to be someone well-known for something other than their MI. A report about someone who is perhaps prominent (e.g. a business executive) but is unlikely to have appeared except for their MI issues (e.g. suicide) is ‘general public’.
  1. Causes of MI (non personal responsibility)
     1. Illness
     2. Psychosocial
     3. Other
     - *Meaning: mental illness happens for a reason beyond the person’s control; they should receive care and sympathy like anyone with an illness.*
     - Code this as primary when the article is mainly about causes in general, or when a portrayal is mainly about the causal process. Often for portrayals it will be secondary. The vast majority of the time this will be psychosocial, but there may be instances where it’s bio e.g. there is a history of depression in my family (unless they describe the transfer mechanism as psychosocial i.e. my mother’s low mood wore off on me).
     - Psychosocial is more likely to be coded, as almost by definition discussing psychosocial causes humanises those with the disorder. However, stories about MI as an illness (i.e. a ‘brain disease’, something genetic) often won’t be coded, as there is less about the people themselves and hence the *meaning* of illness that something that *happens to someone* is not properly expressed. This will often be neutral.
     - ‘Prone to depression’ suggests an underlying genetic trait; code 2.2.1.
     - If both biological and psychosocial causes/treatment are mentioned, code for both. The mere mention of biology doesn’t necessarily require coding as biological e.g. an article which talks about the evolutionarily adaptive nature of depression might nonetheless suggest that when depression *does* happen, it is due to psychosocial causes; code this as psychosocial. The point of this category is essentially to mirror the competing views of mental illness i.e. is it *primarily* an illness, often with genetic cause (which might nonetheless have immediate psychosocial triggers) or is it *primarily* the result of psychosocial stressors (which might nonetheless have a biological basis).
     - Many articles which are about biological causes are not anti-stigmatizing, so don’t code, and many articles which are about ‘other causes’ might be neutral.
     - For PTSD and Post Natal Depression, only code if something specific about the traumatic event or pregnancy is mentioned; somebody just saying that they suffer PTSD or PND is not enough, even though the immediate cause is always implied.
       - In the case of a soldier suffering PTSD, saying they were a soldier is not enough; we have to have knowledge of particular posting/incident which led to the PTSD.
     - Other generally means environmental, chemical etc.
     - Do note code immediate triggers; causal claim must be something about the origin/development of the MI in the first place.
  2. Recovery from/successful treatment of MI
     1. Pharmaceutical
     2. Psychosocial (lifestyle or psychotherapy)
     3. Not specified
     - *Meaning: it is possible to treat and recover from mental illness.*
     - Again, code this as primary when the article is mainly about recovery, whether on an individual or general level. However, likely in many individual stories that recovery would be a secondary element to the primary one of sympathetic portrayal.
     - ‘Recovery from/treatment of MI’ (2.3) should not be coded for an article which simply *mentions* a psychiatric drug in passing (e.g. a report on drugs company profits which briefly mentions high sales of a psychiatric drug); it should be used in articles in which we are given some account of recovery from/successful treatment of MI (this could still be a brief report on a drugs company, provided it specifically notes treatment effects of its drugs).
     - Doesn’t have to specifically described a recovery, but merely note that someone had an MI but doesn’t have it now.
     - This might be the code about an expansion of MH services, provided there’s some comment on their effectiveness e.g. 8/6/09 #14.
     - Alternative medicine goes into pharmaceutical, and physical therapy into psychosocial.
  3. MH promotion
     - *Meaning: people are carrying out activities to improve knowledge and treatment of mental illness.*
     - If it’s about charities, advocating more MH services (unless it’s in the context of a deficiency, hence code 2.6 injustice), and praising the expansion of services.
     - Reports of activities (often by MH charities) trying to improve MH, raise awareness.
       - This can just be a description of the work MH charities normally do; see 8/5/09 #30 and #35 for the difference between when MH charity work is and is not coded.
     - Politicians arguing for better mental health services come here, unless it’s in the context of people actually getting poor service, in which case it’s 2.6 Injustice. This then might appear in the context of an expansion of MH services, in which a charity/politician/SU gives a sense that this was a political/campaigning choice.
     - Descriptions of how to get help if you have an MI, including contact details. Example of ‘how to spot the problem’ is 8/6/09 #17
     - Descriptions and advice relating to the prevention of MI can also be coded as 2.4 if treatment/recovery of MI (2.3) is not applicable.
  4. Stigma faced by people with MI
     - *Meaning: people with mental illness face stigma and deserve sympathy.*
     - Any article about prejudicial attitudes towards people with MI and behaviour *that results from* those attitudes (discrimination).
     - Keep an eye out for this as a secondary element in sympathetic portrayal stories.
  5. Injustice faced by people with MI
- *Meaning: people with mental illness face unfairness resulting from their mental illness and deserve sympathy.*
- 2.5 is about prejudice (i.e. negative attitudes) and the behaviour *that results from* those attitudes, while 2.6 is about people with MI who suffer because they have an MI, but not necessarily because people have negative attitudes (e.g. simply because they’re SUs, and thus they are the ones affected by poor MHS; or because they might be vulnerable to exploitation, though this might often be hopeless victim; review the cases).
- Careful about this. It has to be a sympathetic and sensitive portrayal of the real difficulties faced by someone with MI, and not a portrayal of them as hopeless, helpless, weak victims leading lives of perpetual chaos. Think about whether the person was quoted, whether it looks at them as a whole person, and whether it’s a person/group that readers are likely to sympathize with. For example, if it’s children in a psych hospital, likely to be 2.6, but if it’s an adult male who lives on his own, more likely to be 1.3. On the contrary, it might instead be better coded as hopeless victim (1.3), unusual behaviour (1.4), problem for others (1.2) or pejorative language (1.7). To be 2.6, it has to be a righteous victim.
- The injustice must follow the onset of the MH problem i.e. don’t code for this when an injustice precipitates a MH problem (this likely comes under 2.1).
- Code 2.1 in addition when there’s also life details (in addition to stuff just about the injustice) and the person is portrayed fairly sympathetically i.e. injustice situations might be unusual, hence it’s not a sympathetic portrayal as such.
  1. Prevalence of MI
     - *Meaning: mental illness is widespread; it can happen to anyone.*
     - Statistics about high prevalence of MI, phrases such as ‘MI are widespread’.
     - When an individual(s) sufferer is mentioned to explicitly make the point about prevalence, code as 2.7.
     - If it doesn’t refer to the whole population, must refer to a fairly large group c. 10% of the population or more (e.g. women, men, children, public sector workers [but not subgroups of these] etc.). A more limited group e.g. ‘Many models suffer from eating disorders’ is not sufficient.
